# Assessment of new-onset depression and anxiety associated with COVID-19

**DOI:** 10.1186/s43045-021-00112-w

**Published:** 2021-05-31

**Authors:** Eman Hurissi, Ethar Abu-jabir, Amnah Mohammed, Mashael Mahnashi, Sana Alharbi, Ahmad Alharbi, Ahmed Alnaami, Essam Alameer, Anwar Alahmar, Abdulaziz Alhazmi

**Affiliations:** 1 Faculty of Medicine, Jazan University, Jazan, Saudi Arabia; 2 Department of Medicine, Faculty of Medicine, Jazan University, Jazan, Saudi Arabia; 3 Consultants Psychiatrists, Eradah and Mental Health Hospital, Ministry of Health, Jazan, Saudi Arabia; 4 Department of Microbiology and Parasitology, Faculty of Medicine, Jazan University, Jazan, Saudi Arabia; 5 Medical Research Center, Jazan University, Al Maarefah Rd, Jazan, Jazan, 45142 Saudi Arabia

**Keywords:** SARS-CoV-2, COVID-19, Anxiety, Depression

## Abstract

**Background:**

Psychological disorders are common among individuals who experienced COVID-19. Previous studies have shown that females report higher depression and anxiety than males. The present study aims to test the differences in depression and anxiety between males and females who have experienced COVID-19. This a descriptive, observational, comparative study, among Saudi Arabian population. A total of 686 participants have been recruited. Participants completed an online questionnaire that contains questions about sociodemographic, COVID-19, Generalized Anxiety Disorder (GAD-7) questionnaire, and Patient Health Questionnaire (PHQ-9) to measure anxiety and depression, respectively.

**Results:**

Twenty-six percent of the participants were excluded and our final sample consisted of 507 participants (median age 23; 65% females). Of the final sample, 23% (118) have been previously diagnosed with COVID-19. There is no significant difference in GAD-7 and PHQ-9 scores between COVID-19- positive and COVID-19-negative populations. However, females who have experienced COVID-19 reported significantly higher GAD-7 and PHQ-9 scores compared to males.

**Conclusion:**

The results of our study show that females are significantly at a higher risk for depression and anxiety as a result of COVID-19 infection compared to males. Further epidemiological studies are required for a better understanding of this correlation.

## Background

The coronavirus disease 2019 (COVID-19) declared as a global pandemic on March 11, 2020, by the World Health Organization (WHO). COVID-19 is a respiratory illness that was first identified amid an outbreak of respiratory coronavirus 2 (SARS-CoV-2) in Wuhan City [1]. The most prevalent symptoms associated with COVID-19 are fever, cough, fatigue, loss of smell and taste, and shortness of breathing [2]. Prior studies reported that COVID-19 patients may experience mental health conditions such as anxiety and depression [3]. Coronaviruses are neurotropic that can invade the brain through the olfactory neural pathway or a significant inflammatory response that can cause peripheral and central nervous system (CNS) manifestations [4]. Studies showed that patients affected with severe acute respiratory syndrome (SARS), which is caused by SARS CoV-1, reported a wide range of psychiatric complications, including adjustment-related anxiety and depression [5]. Those complications may last after the infection as patients with SARS found to have post-traumatic stress disorder (54%), depression (39%), panic disorder (32.5%), and obsessive-compulsive disorder (15.6%), 50 months post-infection [6]. For COVID-19, studies showed that 20 to 40% of patients showed neuropsychiatric symptoms and delirium during acute infection [7]. For the psychiatric symptoms in acutely infected patients with COVID-19, anxiety and depressive symptoms were reported in 35% and 28% of patients, respectively [7]. After discharge, studies showed that COVID-19 patients might manifest persistent psychiatric illnesses, including anxiety and depressive disorders [8, 9]. Furthermore, depression and anxiety exhibited in COVID-19 patients were most closely associated with a loss of smell and taste rather than other symptoms of severe indicators of COVID-19 such as shortness of breath, cough, or fever [10]. The prognosis of COVID-19 could be worsened by psychological distress and depression, which may negatively affect the patient’s immune system response [11] and COVID-19 patients may experience persistent depression even after the outbreak of infectious diseases [12].

Many surveys have also shown that women are at a greater risk of psychological problems than men within the Saudi population. The latest national survey in Saudi Arabia (The Saudi National Mental Health Survey) showed that the prevalence of any anxiety disorder was 26% in females and 20% in males. It has also shown that the prevalence of any mood disorder is 12% in females and 7% in males. Females have a higher prevalence of any lifetime mental disorder than males (36% vs. 33%) and a higher prevalence of comorbid mental disorders (18% vs 12%) [13]. Those differences also translate to different rates in the total burden of depressive and anxiety disorders between males and females. Among the leading cause of burden of disease measured in disability-adjusted life years (DALYs), depressive disorders are the 7th leading cause in males (at a rate of 718 DALYs per 100,000) and the 5th leading cause in females (at a rate of 371 DALYs per 100,000). Anxiety disorders are the 16th leading cause in males (at a rate of 718 DALYs per 100,000) and the 10th leading cause in females (at a rate of 630 DALYs per 100,000) [14].

Given those differences, we aim in this study to explore if the onset of COVID-19 might have differential outcomes between males and females in the Saudi population.

## Methods

### Study design and participants

This is a descriptive, observational, comparative study, using Google Form platform contained validated online survey among Saudi Arabia population which includes all adult males and females during or post COVID-19 infection and compared to the non-COVID-19 population as a control group. We calculated the sample size needed to estimate mental disorder prevalence rates with a confidence level of 95% and a 5% margin of error as n = 377, given the expected response rate of 50%. Data were gathered from this group using validated online questionnaires (PHQ-9; Patient Health Questionnaire-9 and GAD-7; General Anxiety Disorder-7) [15], in addition to questions about demographic data and medical history (summarized in Table 1). This study was conducted between 01/10/2020 and 31/01/2021. Ethical approval was granted from the Scientific Committee at the faculty of Medicine, Jazan University, and informed consent has been obtained from all participants.

**Table 1:** Questions about demographic data and medical history. *PHQ-9* Patient Health Questionnaire-9 and *GAD-7* General Anxiety Disorder-7 that are included in the questionnaire

Questions	Options			
1. Age	Open question			
2. Gender	Male, female			
3. Marital status	Single, married, divorced, widower/ widow			
4. For females: are you pregnant?	Yes, No			
5. Nationality	Saudi, Non-Saudi			
6. Job-status	Student, government sector employee, private sector employee, freelancer, retired, unemployed			
7. Have you been diagnosed with COVID-19?	Yes, No			
8. Do you know anyone of your relatives or friends is infected with COVID-19?	Yes, No			
9. Have you been diagnosed with any psychiatric disorder and are you using medication for it?	Yes, No			
10. Over the last two weeks, how often have you been bothered by any of the following problems? (Or four weeks after you've been diagnosed with COVID-19)				
GAD-7	Not all	Several days	More than half the days	Nearly every day
1. Feeling nervous, anxious or on edge?	0	1	2	3
2. Not being able to stop or control worrying?	0	1	2	3
3. Worrying too much about different things?	0	1	2	3
4. Trouble relaxing?	0	1	2	3
5. Being so restless that it is hard to sit still?	0	1	2	3
6. Becoming easily annoyed or irritable?	0	1	2	3
7. Feeling afraid as if something awful might happen?	0	1	2	3
11. Over the last two weeks, how often have you been bothered by any of the following problems? (Or four weeks after you've been diagnosed with COVID-19, if you've already been infected)				
PHQ-9	Not all	Several days	More than half the days	Nearly every day
1. Little interest or pleasure in doing things?	0	1	2	3
2. Feeling down, depressed, or hopeless?	0	1	2	3
3. Trouble falling or staying asleep, or sleeping too much?	0	1	2	3
4. Feeling tired or having little energy?	0	1	2	3
5. Poor appetite or overeating?	0	1	2	3
6. Feeling bad about yourself - or that you are a failure or have let yourself or your family down?	0	1	2	3
7. Trouble concentrating on things, such as reading the newspaper or watching television?	0	1	2	3
8. Moving or speaking so slowly that other people could have noticed? Or the opposite - being so fidgety or restless that you have been moving around a lot more than usual?	0	1	2	3
9. Thoughts that you would be better off dead, or of hurting yourself in some way?	0	1	2	3

### Statistical analysis

Descriptive data were analyzed using suitable tool such as t-test, chi-squared test, ANOVA test, and binary logistic regression for univariate analyses and multivariate analysis. *p*-value less than 0.05 is considered statistically significant. The analysis of data was performed using SPSS Statistic v23.0

## Results

The data were collected from 686 Saudi adults through a self-administrated electronic questionnaire. Our final sample consisted of 507 participants after the exclusion of 179 participants. The excluded participants were either pregnant women (3%), job seekers (11%), who have a history of mental disorders (8%), or those who are less than 18 years (3%).

The age of participants ranged from 18 to 70 years old with a median of 23 years and a standard deviation of 8 (Table 2). 35% of participants were male while 65% are females. 27% of participants were married while 71% were single. 65% of participants are students and 33% are employed while 1% are retired. Regarding the COVID-19-positive and COVID-19-negative participants, 23% of participants reported having been diagnosed with COVID-19.

**Table 2:** Descriptive data included all participants in our analytic sample

Total participants =507	
Age (year)	
Median (SD: range)	23 (8: 18 to 70)
Gender	
Male	35%
Female	65%
Marital status	
Single	71%
Married	27%
Divorce	2%
Occupation	
Student	65%
Governmental sector	22%
Private sector	11%
Retired	1%
COVID-19-positive cases	23%
GAD-7	
Average (SD)	9 (7)
PHQ-9	
Average (SD)	10 (9)

*GAD-7* General Anxiety Disorder-7, *PHQ* Patient Health Questionnaire-9, *SD* standard deviation

Scores of PHQ-9 ranged from 0 to 27 (the mean is 10.1 and the standard deviation is 6.38). Depression prevalence in our sample was 45%, 54.4% in females and 27.8% in males. Scores of GAD-7 ranged from 0 to 21 (the mean of 9.13 and the standard deviation is 5.20). Anxiety prevalence in our sample was 42.4%, 50.8% in females and 26.7% in males.

Regarding the overall effect of COVID-19 infection on depression and anxiety, we compared the scores of the two populations using an independent samples t-test and did not find a significant difference in GAD-7 and PHQ-9 scores between COVID-19 positive population and COVID-19 negative populations (all *p*s > .1; Table 3).

**Table 3:** Statistical comparisons between GAD-7 and PHQ-9 among COVID-19 positive and COVID-19 negative

Measure	COVID-19 negative (*N*=389)	COVID-19 positive (*N*=118)	Student’s t-test *p*-value
GAD-7	9.33 (5.10)	8.45 (5.49)	0.106
PHQ-9	10.03 (6.27)	10.42 (6.73)	0.553

*GAD-7* General Anxiety Disorder-7, *PHQ* Patient Health Questionnaire-9, *SD* standard deviation

We then asked if those differences in PHQ-9 and GAD-7 scores are different within males and females. To answer this question, we first conducted an analysis of variance (ANOVA) with PHQ-9 scores as the dependent variable while the independent variables were gender and COVID-19 infection (Table 4). We observed an overall effect of gender [F(1, 503)= 39.24, *p*<0.001], no effect of COVID-19 infection [F(1, 503)= 0.74, *p*=0.391], and most noticeably, a significant interaction between gender and COVID-19 infection [F(1, 503) = 4.443, *p*=0.036]. To understand this interaction, we conducted a series of post hoc Student’s t-tests with all possible pairwise comparisons between COVID-19-positive and COVID-19-negative population and gender. Post hoc t-tests show that PHQ-9 scores are significantly lower in females who are COVID-19 negative compared to the females who are COVID-19 positive [t(503) = −2.318, *p*=0.021]. Furthermore, this difference is not observed in men as PHQ-9 scores are not significantly different within males who are COVID-19 positive from those who are COVID-19 negative [t(503) = 0.813, *p*=0.417]. Those outcomes show that PHQ-9 is affecting females who had COVID-19 more than males who had COVID-19. For the GAD-7 scores, we also conducted ANOVA analysis with GAD-7 scores as the dependent variable and with gender and COVID-19 infection as independent variables (see Table 5). The model shows a significant overall effect of gender [F(1,503) = 38.31, *p*<0.001], a nonsignificant effect of COVID-19 infection [F(1,503)=1.72, *p*=0.191] and a trending effect of the interaction between gender and COVID-19 [F(1,503) = 3.10, *p*=0.079]. Since the effect is not significant, we did not run any post hoc analyses (Table 5).

**Table 4:** Analysis of variance (ANOVA) of the effect of gender and COVID-19 infection on the scores of PHQ-9

	Sum of squares	df	Mean square	F	*p*
Gender	1485.2	1	1485.2	39.239	< .001
COVID-19	27.9	1	27.9	0.737	0.391
Gender x COVID-19	168.2	1	168.2	4.443	0.036
Residuals	19,038.8	503	37.9		

*PHQ* Patient Health Questionnaire-9, *df* degrees of freedom

**Table 5:** Analysis of variance (ANOVA) of the effect of gender and COVID-19 infection on the scores of GAD-7

	Sum of squares	df	Mean square	F	*p*
Gender	958.7	1	958.7	38.31	< .001
COVID-19	43.0	1	43.0	1.72	0.191
Gender x COVID-19	77.7	1	77.7	3.10	0.079
Residuals	12,586.9	503	25.0		

*GAD-7* General Anxiety Disorder-7, *df* degrees of freedom

## Discussion

This study aims to assess the new-onset depression and anxiety disorders in COVID-19 patients in Saudi Arabia during the pandemic. In this study, we sought the association between COVID-19 infection and new-onset mental health disorders such as depression and anxiety in COVID-19 patients, especially females. Though there is no significant difference in the prevalence of anxiety and depression among COVID-19-positive or negative-populations reported in this study, we found the females are more affected by COVID-19 than males in depression scores and to a moderate extent in the anxiety scores. While we cannot identify the underlying causal mechanisms, previous studies reported that COVID-19 infection, or coronaviruses infections, may increase the risk of anxiety and depression. For example, in a systematic search that included 3559 studies, it was found that depressed mood and anxiety were common symptoms among patients admitted to the hospital for SARS or MERS during the acute illness or the post-illness stage [7]. In another study that analyzed 69 million health records from over 62,000 people diagnosed with COVID-19, it showed that 6% of COVID-19 patients experienced mental health disorders such as depression and anxiety within 3 months of diagnosis compared to 3.4% of non-COVID-19 patients [16]. From a molecular biology perspective, different pathogenetic mechanisms have been described as central nervous system affection by SARS-CoV-2. However, the exact pathogenetic mechanism associated with neuropsychiatric symptoms in COVID-19 patients is currently unknown and needs to be elucidated. Some previous reports highlighted the ability of Coronavirus to penetrate the brain through the olfactory canal, retrograde neural pathway, or by induce a significant inflammatory response through activation of the inflammatory pathway and release cytokines into the body. The blood-brain barrier is likely to be damaged as a result of increased pro-inflammatory factors in the bloodstream making functional damage possible [4]. Moreover, different pathways of indirect infection of the CNS have been suggested, including host immune response against the virus, pre-existence of acute toxic encephalopathy, or as a side effect of COVID-19 medical treatment [4].

In the current study, we also found that females with COVID-19 are at high risk for mental disorders compared with males (Tables 4 and 5). This result is consistent with many other studies. For example, in one follow-up study, after 1 month of hospital treatment, which was done on 402 adult patients who were diagnosed with COVID-19 (66% male, mean age 58), it has been found that 42% of patients reported anxiety while 31% of patients reported depression (among many other mental conditions) and, furthermore, females have experienced more anxiety and depression than males [17]. Another study on 76 patients who were quarantined in fever-isolation wards with suspected COVID-19 to investigate anxiety and depression has found that female patients are more likely to experience depression and anxiety than male [18]. Another study investigated the gender differences in depression, anxiety, and the associated factors during the COVID-19 epidemic among Chinese social media users. The findings suggested that the increased prevalence of depression and anxiety during the COVID-19 epidemic among the Chinese population. Furthermore, females showed more severe anxiety symptoms than males [19]. Another study, conducted in Kuwait, aimed to assess the prevalence of anxiety and depression symptoms in Kuwaiti nationals and expats. The study’s secondary objective was to highlight the association between physical activity (PA) engagement and sociodemographic characteristics, with mental health disorders such as anxiety and depression during the COVID-19 pandemic. A web-based cross-sectional survey was used to examine the sociodemographic characteristics and PA engagement and generalized anxiety and depression symptoms. The results showed that anxiety was reported in more than 50% of the sample, and depressed mood reported in approximately 60% of the sample during COVID-19 outbreaks. Moreover, women and younger individuals with lower PA and education were more likely to develop anxiety symptoms while depressive symptoms were more prevalent among women, people with lower PA and education, elderly, and married people [20]. Overall, the patterns in this study are largely consistent with those reported in the literature. Notably, the prevalence of anxiety and depression in our study was relatively higher than the national rates [14]. The national rates were based on Saudi National Health and Stress Survey which has been conducted many years before the coronavirus outbreak while our survey has been conducted amid the outbreak, a likely factor that inflated anxiety and depression. While we do not know of any current estimates of the prevalence of anxiety and depression disorders, the prevalence in our sample is similar to other studies conducted in similar populations. For example, in a study conducted in Kuwait [20], anxiety prevalence was reported to be 50% while depression was reported to be 60% during the outbreak [18, 20].

This study has many limitations. As an online survey, there is potentially self-selection bias against those who did not have access to the Internet, and individuals who were more concerned about their mental health might have been more likely to participate in the study. Moreover, we used self-reported scales to assess anxiety and depression because it was convenient especially during the pandemic and social distancing measures, and that might lead to recall bias. Furthermore, the results of this study cannot be generalized to other populations because of possible sampling error. However, to the best of our knowledge, this is the first study that investigated the prevalence of new-onset mental health symptoms (i.e., symptoms of depression and anxiety) by standardized rating scales among the Saudi population during the COVID-19 pandemic. Our findings may provide more helpful recognition of high-risk populations and framework design for population-specific psychological crisis management. Furthermost, this study calls attention to the importance of early psychological therapeutic intervention because the prognosis of COVID-19 could be worsened by psychological distress and depression, which may negatively affect the patient’s immune system response.

## Conclusion

In conclusion, we found very high levels of depression and anxiety among the sample. The high levels of depression and anxiety may have masked the differences between those with or without COVID-19. However, we found that females diagnosed with COVID-19 had a higher prevalence of depression and anxiety compared to males diagnosed with COVID-19. The impact of COVID-19 on psychological wellbeing is highly significant, especially in females. Thus, future epidemiological studies, as well as systematic reviews pooling the evidence, are needed to adapt public health interventions.

## Data Availability

The data supporting the findings in this study are confidentially available from the corresponding author upon reasonable request.

## Abbreviations

GAD-7: Generalized Anxiety Disorder
PHQ-9: Patient Health Questionnaire
COVID-19: Coronavirus disease 2019
SARS: Severe acute respiratory syndrome
WHO: World Health Organization
CNS: Central nervous system
DALYs: Disability-adjusted life years
ANOVA: Analysis of variance
PA: Physical activity

## References

[1] Listings of WHO’s response to COVID-19 [Internet]. Who.int. [cited 2021 May 12]. Available from: https://www.who.int/news/item/29-06-2020-covidtimeline

[2] Home - johns Hopkins Coronavirus resource center [Internet]. Jhu.edu. [cited 2021 May 12]. Available from: https://coronavirus.jhu.edu/covid-19-basics/faq

[3] Canady VA (2020). Study finds COVID-19 survivors exhibit MH signs one month after treatment. Ment Health Wkly..

[4] Desforges M, Le Coupanec A, Dubeau P, Bourgouin A, Lajoie L, Dubé M (2019). Human coronaviruses and other respiratory viruses: Underestimated opportunistic pathogens of the central nervous system?. Viruses..

[5] Cheng SK-W, Tsang JS-K, Ku K-H, Wong C-W, Ng Y-K (2004). Psychiatric complications in patients with severe acute respiratory syndrome (SARS) during the acute treatment phase: a series of 10 cases. Br J Psychiatry..

[6] Lam MH-B, Wing Y-K, Yu MW-M, Leung C-M, Ma RCW, Kong APS (2009). Mental morbidities and chronic fatigue in severe acute respiratory syndrome survivors: long-term follow-up: Long-term follow-up. Arch Intern Med..

[7] Rogers JP, Chesney E, Oliver D, Pollak TA, McGuire P, Fusar-Poli P, Zandi MS, Lewis G, David AS (2020). Psychiatric and neuropsychiatric presentations associated with severe coronavirus infections: a systematic review and meta-analysis with comparison to the COVID-19 pandemic. Lancet Psychiatry..

[8] Galea S, Merchant RM, Lurie N (2020). The mental health consequences of COVID-19 and physical distancing: The need for prevention and early intervention: The need for prevention and early intervention. JAMA Intern Med..

[9] Siddaway AP (2020). Multidisciplinary research priorities for the COVID-19 pandemic. Lancet Psychiatry..

[10] Marlene M. Speth, Thirza Singer‐Cornelius, Michael Oberle, Isabelle Gengler, Steffi J. Brockmeier, Ahmad R. Sedaghat, (2020) Mood, Anxiety and Olfactory Dysfunction in ‐19: Evidence of Central Nervous System Involvement?. The Laryngoscope 130(11):2520-2525 10.1002/lary.28964 PMC7361512 32617983

[11] L BE. The immune system, depression and the action of antidepressants [Internet. Vol. 25. 2001. p. 767–780. 10.1016/s0278-5846(01)00155-5 11383977

[12] Ma Y-F, Li W, Deng H-B, Wang L, Wang Y, Wang P-H, Bo HX, Cao J, Wang Y, Zhu LY, Yang Y, Cheung T, Ng CH, Wu X, Xiang YT (2020). Prevalence of depression and its association with quality of life in clinically stable patients with COVID-19. J Affect Disord..

[13] Altwaijri YA, Al-Subaie AS, Al-Habeeb A, Bilal L, Al-Desouki M, Aradati M (2020). Lifetime prevalence and age-of-onset distributions of mental disorders in the Saudi National Mental Health Survey. Int J Methods Psychiatr Res..

[14] GBD 2017 Disease and Injury Incidence and Prevalence Collaborators (2018). Global, regional, and national incidence, prevalence, and years lived with disability for 354 diseases and injuries for 195 countries and territories, 1990-2017: a systematic analysis for the Global Burden of Disease Study 2017. Lancet.

[15] Sawaya H, Atoui M, Hamadeh A, Zeinoun P, Nahas Z (2016). Adaptation and initial validation of the Patient Health Questionnaire - 9 (PHQ-9) and the Generalized Anxiety Disorder - 7 Questionnaire (GAD-7) in an Arabic speaking Lebanese psychiatric outpatient sample. Psychiatry Res..

[16] Taquet M, Luciano S, Geddes JR, Harrison PJ (2021) Bidirectional associations between COVID-19 and psychiatric disorder: retrospective cohort studies of 62 354 COVID-19 cases in the USA. The Lancet Psychiatry 8(2):130-140 10.1016/S2215-0366(20)30462-4 PMC7820108 33181098

[17] Mazza MG, De Lorenzo R, Conte C, Poletti S, Vai B, Bollettini I, Melloni EMT, Furlan R, Ciceri F, Rovere-Querini P, Benedetti F (2020) Anxiety and depression in COVID-19 survivors: Role of inflammatory and clinical predictors. Brain, Behavior, and Immunity 89:594-600 10.1016/j.bbi.2020.07.037 PMC7390748 32738287

[18] Li X, Dai T, Wang H, Shi J, Yuan W, Li J, Chen L, Zhang T, Zhang S, Kong Y, Yue N, Shi H, He Y, Hu H, Liu F, Yang C (2020). Clinical analysis of suspected COVID-19 patients with anxiety and depression. Zhejiang Da Xue Xue Bao Yi Xue Ban..

[19] Hou F, Bi F, Jiao R, Luo D, Song K (2020). Gender differences of depression and anxiety among social media users during the COVID-19 outbreak in China:a cross-sectional study. BMC Public Health..

[20] Alsharji KE. Anxiety and depression during the COVID-19 pandemic in Kuwait: the importance of physical activity. Middle East Curr Psychiatr [Internet]. 2020;27(1). Available from: 10.1186/s43045-020-00065-6

